# Sexual behavior and its influencing factors among Uruguayan adolescents: a cross-sectional study

**DOI:** 10.1016/j.xagr.2025.100538

**Published:** 2025-06-19

**Authors:** Tonmoy Alam Shuvo, Kabir Hossain, Arifur Rahman

**Affiliations:** Department of Statistics, Noakhali Science and Technology University, Noakhali, Bangladesh

**Keywords:** adolescent health, early sexual initiation, influencing factors, sexual behavior, Uruguayan adolescents

## Abstract

**Background:**

This study aimed to investigate the factors influencing adolescent sexual behavior in Uruguay, with a focus on socio-demographics, substance use, psychological distress, and protective factors.

**Methods:**

We analyzed data from the 2019 Uruguay Global School-based Student Health Survey (GSHS). Chi-square tests identified variables significantly associated with sexual behavior. Mixed-effects logistic regression models estimated odds ratios, and a fully adjusted model provided adjusted odds ratios for all significant factors. All analyses were performed using R programming.

**Result:**

Our analysis included a total of 2495 participants. Older adolescents (≥15 years) had a significantly higher likelihood of engaging in sexual behavior (AOR: 3.69, 95% confidence intervals [CI]: 2.92–4.66). Tobacco (AOR: 2.60, 95% CI: 1.91–3.55) and alcohol use (AOR: 2.09, 95% CI: 1.71–2.56) were strong risk factors, along with alcohol-related behavioral issues (AOR: 2.08, 95% CI: 1.65–2.62). Anxiety (AOR: 1.51, 95% CI: 1.09–2.08) and involvement in physical fights (AOR: 1.84, 95% CI: 1.42–2.40) increased the odds, while loneliness showed a protective effect (AOR: 0.62, 95% CI: 0.46–0.83). Parental bonding (OR: 0.54, 95% CI: 0.45–0.65), attachment (OR: 0.70, 95% CI: 0.59–0.82), and supervision (OR: 0.64, 95% CI: 0.54–0.77) showed significant effects before adjustment, but after adjustment, they became insignificant. Sedentary behavior reduced the likelihood, while truancy increased the possibility of sexual behavior among adolescents.

**Conclusion:**

Interventions should aim to reduce adolescent sexual risk behaviors by promoting healthy practices and addressing factors such as substance use, mental health, and parental involvement to encourage safer sexual behaviors.

**AJOG Global Reports at a Glance**

**Why was this study conducted?**

This study investigates factors influencing adolescent sexual behavior in Uruguay, focusing on socio-demographics, substance use, psychological distress, and protective factors.

**Key findings**

Older adolescents, tobacco and alcohol use, anxiety, and physical fighting were significant risk factors for sexual behavior. Loneliness showed a protective effect. Parental bonding, attachment, and supervision appeared protective in unadjusted models but lost significance after adjustment.

**What does this add to what is known?**

This study highlights key risk and protective factors shaping adolescent sexual behavior in Uruguay, offering evidence to inform targeted interventions focused on substance use, mental health, and family involvement.

## Introduction

The period of adolescence, beginning around age 10 and continuing into early adulthood, is marked by swift and dynamic growth across physical, cognitive, emotional, and social domains. This period usually involves curiosity, experimentation, and a tendency toward risk-taking behaviors.^1^ Risky sexual behavior includes a variety of hazardous behaviors such as premarital sex, multiple sexual partners, and unprotected sex.^2^ According to reports, engaging in such risky sexual behaviors can have negative health effects, such as sexually transmitted infections (STIs), unintended pregnancy, psychological distress, and long-term health problems.^3^^,^^4^ Based on recent global data, 6.9% of adolescents aged 12 to 15 reported having ever engaged in sexual activity, with boys at 10.0% and girls at 4.2%.^5^ In low- and middle-income countries, 16.2% of adolescents aged 13 to 17 reported having sexual intercourse. The Americas had the highest regional prevalence (30.5%), followed by Africa (28.6%), the Eastern Mediterranean (10.9%), South-East Asia (9.6%), and the Western Pacific (8.0%).^6^ Adolescent sexual activity has been on the rise in Uruguay, where 27.5% of teenagers between the ages of 12 and 15 engage in sexual activity. Consequently, this rise in sexual activity is associated with high-risk behaviors, such as 30.4% having sex before the age of 14, 45.7% reporting multiple sex partners, 15.6% not using condoms during sex, and 34.1% not using birth control methods. Further investigation in this area is necessary, as neighboring countries like Brazil and Argentina also report rising rates of adolescent sexual behavior.^6^^,^^7^

The rate of youth engaging in sexual activity increased with age for both genders, with males having a higher rate than females.^8^, ^9^, ^10^ Similarly, living in a rural area raised the likelihood of having sex earlier in life.^8^ Adolescents with higher levels of education were less likely than their less educated peers to undergo early sexual initiation (ESI), suggesting that education acts as a protective factor.^11^ Higher socioeconomic status was linked to lower levels of sexual activity, whereas joblessness and being in the lowest wealth quintile were associated with early sexual activity.^8^^,^^9^^,^^11^ Academic achievement and parental expectations significantly reduced sexual activity, likely due to a greater emphasis on academics, long-term goals, and a desire to live up to parental expectations.^9^ Further demonstrating the importance of family relationships, maternal support, and good parental communication were significant predictors of decreased severity of present and future compulsive sexual behavior.^12^ Additionally, religiosity served as a protective factor because those who practiced their religion more were less likely to have sex, which is frequently motivated by moral or spiritual beliefs. Any sexual conduct and mental health factors were related, as adolescents who did not have suicidal thoughts were less likely to have sex, which may indicate that they had better mental health and more effective coping mechanisms.^9^ On the other hand, the connection between sexual activity and suicidal intentions or attempts shows that such behaviors may be accompanied by underlying emotional distress and mental health issues.^13^ Substance use, such as smoking and alcohol intoxication, was significantly associated with risky sexual behaviors, and adolescents were more likely to engage in these behaviors if their parents also used drugs.^14^^,^^15^

While many studies have been conducted in various regions, research focusing specifically on Uruguay remains scarce. Most available literature in Latin America tends to generalize findings across countries, overlooking national differences in culture, education, and healthcare systems. In Uruguay, there is a lack of recent and detailed understanding of the factors that influence adolescent sexual behaviors. Although adolescent health has been studied to some extent in Uruguay,^16^ the specific behavioral, psychosocial, and demographic influences on sexual health remain underexplored.

It is important to gain a better understanding of how family, peers, education, substance use, and socioeconomic factors shape sexual behavior among adolescents in Uruguay. Without this understanding, it is challenging to design effective, evidence-based interventions tailored to their needs. Given these gaps, our study provides timely and context-specific evidence. This study aims to explore the sexual behavior of adolescents in Uruguay and determine the factors that influence it. It focuses on examining how individual characteristics, emotional and psychological well-being, social interactions, and family dynamics are associated with adolescents’ sexual decisions. The research also considers how lifestyle patterns and school-related behaviors contribute to these outcomes. Through a comprehensive analysis, the study seeks to uncover the complex interplay of risks and protective influences shaping adolescent sexual behavior. The findings aim to support the development of informed interventions and policies to promote healthier behaviors among youth in Uruguay. This study sheds light on the crucial factors influencing adolescent sexual behavior in Uruguay, offering important information for policymakers, educators, and healthcare providers. This study addresses a key gap to guide targeted adolescent health interventions.

## Methods

### Data information

We analyzed data from the 2019 Uruguay Global School-based Student Health Survey (GSHS) to investigate the relationships between various factors and sexual behavior.^17^ The GSHS is a joint surveillance initiative aimed at assisting countries in evaluating behavioral risk and protective factors across 10 key areas among adolescents.^18^ The GSHS is a school-based, paper-based survey that collects data through self-administered questionnaires. Countries design their questionnaires by combining standardized core and expanded questions with additional items tailored to their specific needs. A two-stage cluster sampling method was used to gather data representative of students in grades CB-3 BD in Uruguay for the 2019 GSHS. In the first stage, 66 schools were chosen with a probability proportional to their enrollment size. In the second stage, classes were randomly selected, and all students within those classes were eligible to participate. The response rates for the survey were as follows: 94% for schools, 69% for students, and an overall response rate of 64%.^17^ The World Health Organization and the US Centers for Disease Control carried out all aspects of data processing, including scanning, cleaning, editing, and weighting.

### Variable selection

Variables were selected to represent individual, behavioral, emotional, social, and environmental domains, based on existing literature and the availability of data in the dataset. Studies highlighting key risk and protective factors guided the inclusion of variables across multiple domains. Variables associated with early or risky sexual behavior in international and regional research were given attention. Relevance to the research objective and potential impact on the outcome were taken into consideration. Our main focus was on key domains influencing adolescent sexual behavior, including sociodemographic factors, substance use, psychosocial distress, and protective factors.

### Dependent variable

Student’s history of sexual intercourse (yes, no).

### Independent variables

The independent variables in the analysis included gender (male, female), age (≤14, ≥15), hunger (yes, no), tobacco use (yes, no), alcohol use (yes, no), alcohol behavior issue (yes, no), loneliness (yes, no), anxiety (yes, no), suicide ideation (yes, no), suicide attempt (yes, no), physically attacked (yes, no), bullied (yes, no), physical fight (yes, no), peer support (yes, no), parental supervision (yes, no), parental attachment (yes, no), parental bonding (yes, no), sedentary behavior (yes, no), close friend (yes, no), truancy (yes, no). Table 1 represents the description of the variables.

**Table 1:** Description of the variables

Variable	Description
**Socio-demographics**	
Age	Age of the student.
Gender	Gender of the student.
Hunger	Often went hungry due to insufficient food in the past 30 d.
**Sexual behavior**	
Sexual intercourse	Student’s history of sexual intercourse.
**Substance use**	
Tobacco use	Used tobacco at least once in the past 30 d.
Alcohol use	Drank alcohol at least once in the past 30 d.
Alcohol behavior issue	Faced trouble or missed school due to alcohol use.
**Psychosocial distress**	
Loneliness	Often felt lonely in the past year.
Suicide attempt	Attempted suicide in the past year.
Suicide ideation	Considered suicide in the past year.
Physical fight	Involved in a physical fight in the past year.
Bullying victimization	Experienced bullying at school in the past year.
Anxiety	Felt overly worried and unable to sleep at night in the past year.
Physically attacked	Experienced physical attack within the past year.
**Protective factors**	
Close friends	Had close friends.
Peer support	Reported helpful peers in school in the past 30 d.
Parental supervision	Parents checked their homework regularly in the past 30 d.
Parental attachment	Parents understood their problems and worries in the past 30 d.
Parental bonding	Parents knew what they did in their free time in the past 30 d.
Sedentary behavior	Spent 3+ h daily on sedentary activities in the past 30 d.
**Others**	
Truancy	Missed school without permission in the past 30 d.

### Statistical analysis

We initially performed a chi-square test for each independent variable against the dependent variable to identify the significant variables. Subsequently, we fitted separate mixed-effect logistic regression models for each significant independent variable and the dependent variable. This approach allowed us to obtain the odds ratios (OR) for each significant independent variable. We also fitted a comprehensive mixed-effect logistic regression model that included all significant independent variables in the chi-square test to determine their adjusted OR (AOR). Mixed effects logistic regression is used for GSHS survey data because the data are hierarchical or clustered; for example, students are grouped within schools or regions. This clustering creates correlations among observations within the same group, which violates the independence assumption of standard logistic regression. Mixed effects models include random effects to account for variability between clusters. This leads to more accurate estimates and correct standard errors, resulting in more reliable conclusions about the relationship between predictors and the binary outcome, such as sexual behavior. All models used in the analysis are provided in the Supplementary File. Analyses and computations were executed using R programming.

## Results

We utilized datasets comprising 2495 participants, including 1372 females and 1123 males. Table 2 illustrates the relationship between sexual intercourse and various socio-demographics, substance use, psychosocial distress, protective factors, and other factors among adolescents.

**Table 2:** Cross-classification of sexual intercourse by several factors

		Sexual intercourse		
Variable		No (%)	Yes (%)	*P* value
Socio-demographics				
Gender	Male	625 (52.30)	498 (47.70)	.554
	Female	763 (53.72)	609 (46.28)	
Age	≤14	636 (77.83)	170 (22.17)	<.001
	≥15	752 (42.94)	937 (57.06)	
Hunger	No	1368 (53.32)	1081 (46.68)	.127
	Yes	20 (40.77)	26 (59.23)	
Substance use				
Tobacco	No	1313 (58.85)	824 (41.15)	<.001
	Yes	75 (19.91)	283 (80.09)	
Alcohol	No	855 (70.78)	314 (29.22)	<.001
	Yes	533 (38.97)	793 (61.03)	
Alcohol behavior issue	No	1195 (62.95)	630 (37.05)	<.001
	Yes	193 (28.27)	477 (71.73)	
Psychosocial distress				
Loneliness	No	1180 (53.85)	907 (46.15)	.044
	Yes	208 (49.27)	200 (50.73)	
Anxiety	No	1269 (54.64)	935 (45.36)	<.001
	Yes	119 (41.68)	172 (58.32)	
Suicide ideation	No	1179 (55.32)	848 (44.70)	<.001
	Yes	209 (43.53)	259 (56.47)	
Suicide attempt	No	1266 (54.22)	954 (45.78)	<.001
	Yes	122 (44.12)	153 (55.88)	
Physical fight	No	1215 (56.55)	830 (43.45)	<.001
	Yes	173 (36.39)	277 (63.61)	
Bullied	No	1160 (53.85)	888 (46.15)	.034
	Yes	228 (49.50)	219 (50.50)	
Physically attacked	No	1253 (54.96)	911 (45.04)	<.001
	Yes	135 (50.10)	196 (59.90)	
Protective factors				
Peer support	No	464 (52.16)	387 (47.84)	.448
	Yes	924 (53.57)	720 (46.43)	
Parental attachment	No	631 (48.74)	608 (51.26)	<.001
	Yes	757 (57.51)	499 (42.49)	
Parental supervision	No	862 (48.87)	811 (51.13)	<.001
	Yes	526 (61.85)	296 (38.15)	
Parental bonding	No	353 (43.03)	428 (56.97)	<.001
	Yes	1035 (57.86)	679 (42.14)	
Close friend	No	78 (58.58)	49 (41.42)	.209
	Yes	1310 (52.78)	1058 (47.22)	
Sedentary behavior	No	477 (49.99)	423 (50.01)	.042
	Yes	911 (54.81)	684 (45.19)	
Others				
Truancy	No	1085 (61.05)	617 (38.95)	<.001
	Yes	303 (36.63)	490 (63.37)	

The *P* value indicates the result of the Chi-square test.

*Shuvo. Sexual behavior and its influencing factors among Uruguayan adolescents. AJOG Glob Rep 2025.*

Gender did not show a significant association, with nearly equal percentages of males (47.70%) and females (46.29%) reporting sexual intercourse. Age was a significant determinant, with older adolescents (≥15 years) reporting a much higher prevalence (57.05%) compared to younger adolescents (≤14 years, 22.19%). Hunger was not significantly associated with sexual behavior, although a slightly higher prevalence was noted among those who experienced hunger (59.23%).

Substance use strongly influenced sexual behavior, with tobacco use (80.09% vs 41.14%; *P*<.001), alcohol use (61.03% vs 29.22%; *P*<.001), and alcohol behavior issues (71.73% vs 37.05%; *P*<.001) showing significant associations. Psychosocial distress factors like loneliness (*P*=.044), anxiety (58.32% vs 45.36%; *P*<.001), suicide ideation (56.47% vs 44.70%; *P*<.001), and suicide attempts (55.88% vs 45.78%; *P*<.001) were significantly linked to sexual intercourse. Similarly, involvement in physical fights (63.61% vs 43.45%; *P*<.001), bullying (50.50% vs 46.15%; *P*=.034), and being physically attacked (59.90% vs 45.04%; *P*<.001) were positively associated.

Protective factors like parental attachment (42.49% vs 51.76%; *P*<.001), supervision (38.15% vs 51.13%; *P*<.001), and bonding (42.14% vs 56.97%; *P*<.001) significantly reduced the likelihood of sexual behavior. Peer support and having a close friend were not significantly associated. Sedentary behavior showed a weak but significant association, with adolescents reporting higher sexual behavior prevalence (50.00% vs 45.19%; *P*=.042). Finally, truancy (63.37% vs 38.95%; *P*<.001) was significantly linked to sexual intercourse, indicating that missing school is a critical risk factor for adolescent sexual behavior.

Table 3 represents the analysis of factors influencing sexual intercourse, presenting OR, AOR, 95% confidence intervals (CI), and *P* values. Among socio-demographics, adolescents aged ≥15 (OR=4.20, AOR=3.69, *P*<.001) years were significantly more likely to experience the outcome compared to those aged ≤14. The younger (≤14) and older (≥15) groups showed distinct patterns in influencing factors. For the younger group, suicide ideation, alcohol use, and tobacco use strongly increased the likelihood of engaging in sexual intercourse, with alcohol having the highest impact. Parental attachment reduced the likelihood of sexual intercourse in the younger group, but had no significant effect on the older group. For the older group, anxiety and physical fights had a stronger association with sexual intercourse than in the younger group. Alcohol use and tobacco use were weaker factors for the older group compared to the younger group. Interestingly, bullying increased the likelihood of sexual intercourse in the younger group but had the opposite effect in the older group, where it decreased the likelihood. The younger group appeared to be more influenced by external factors such as substance use and bullying, while the older group’s behavior seemed more closely linked to anxiety, physical fights, and other emotional struggles. Truancy had a similar positive effect in both groups, but was slightly stronger for the older group.

**Table 3:** Logistic regression analysis for sexual intercourse

Variable		OR (95% CI)	*P* value	AOR (95% CI)	*P* value
Socio-demographics					
Age	≤14	1.00		1.00	
	≥15	4.20 (3.40, 5.19)	<.001	3.69 (2.92, 4.66)	<.001
Substance use					
Tobacco	No	1.00		1.00	
	Yes	5.70 (4.70, 7.53)	<.001	2.60 (1.91, 3.55)	<.001
Alcohol	No	1.00		1.00	
	Yes	3.85 (3.23, 4.60)	<.001	2.09 (1.71, 2.56)	<.001
Alcohol behavior issue	No	1.00		1.00	
	Yes	4.26 (3.49, 5.21)	<.001	2.08 (1.65, 2.62)	<.001
Psychosocial distress					
Loneliness	No	1.00		1.00	
	Yes	1.22 (0.98, 1.52)	.01	0.62 (0.46, 0.83)	.001
Anxiety	No	1.00		1.00	
	Yes	1.93 (1.49, 2.50)	<.001	1.51 (1.09, 2.08)	.012
Suicide ideation	No	1.00		1.00	
	Yes	1.74 (1.40, 2.15)	<.001	1.11 (0.81, 1.51)	.518
Suicide attempt	No	1.00		1.00	
	Yes	1.69 (1.30, 2.20)	<.001	1.15 (0.80, 1.65)	.454
Physically attacked	No	1.00		1.00	
	Yes	2.18 (1.70, 2.89)		1.28 (0.94, 1.74)	.117
Physical fight	No	1.00		1.00	
	Yes	2.68 (2.14, 3.35)	<.001	1.84 (1.42, 2.40)	<.001
Bullied	No	1.00		1.00	
	Yes	1.37 (1.10, 1.69)	<.001	1.13 (0.88, 1.46)	.338
Protective factors					
Parental bonding	No	1.00		1.00	
	Yes	0.54 (0.45, 0.65)	<.001	0.84 (0.68, 1.06)	.136
Sedentary behavior	No	1.00		1.00	
	Yes	0.80 (0.67, 0.95)	<.001	0.70 (0.57, 0.85)	<.001
Parental attachment	No	1.00		1.00	
	Yes	0.70 (0.59, 0.82)	<.001	0.93 (0.75, 1.14)	.468
Parental supervision	No	1.00		1.00	
	Yes	0.64 (0.54, 0.77)	<.001	0.88 (0.71, 1.09)	.238
Others					
Truancy	No	1.00		1.00	
	Yes	2.65 (2.21, 3.18)	<.001	1.57 (1.28, 1.94)	<0.001

Odds ratios were calculated using mixed-effects logistic regression models with sexual intercourse as the outcome and each independent variable analyzed separately. Adjusted odds ratios were derived from a mixed-effects logistic regression model that included all independent variables, with sexual intercourse as the outcome. The “Others” category included variables that did not fit into the main classifications but were relevant to the analysis and could potentially influence the study outcomes.

*AOR*, adjusted odds ratio; *OR*, odds ratio.

Substance use was a strong predictor, with tobacco users showing the highest odds (OR=5.70, AOR=2.60, *P*<.001), followed by alcohol use (OR=3.85, AOR=2.09, *P*<.001) and alcohol behavior issues (OR=4.26, AOR=2.08, *P*<.001).

Regarding psychosocial distress, loneliness appeared protective in the adjusted model (AOR=0.62, *P*=.001) despite increased odds in the unadjusted model (OR=1.22, *P*=.01). Anxiety remained significant in both models (AOR=1.51, *P*=.012). Suicide ideation and suicide attempts were significant in unadjusted analyses but lost significance after adjustment (AOR=1.11, *P*=.518 and AOR=1.15, *P*=.454, respectively). Being physically attacked and engaging in physical fights were significant in unadjusted models, but only physical fights retained significance after adjustment (AOR=1.84, *P*<.001). Students who were bullied had significantly higher odds (OR=1.37, 95% CI 1.10–1.69, *P*<.001) of engaging in sexual intercourse. However, after adjusting for other variables, this association became insignificant (AOR=1.13, 95% CI 0.88–1.46, *P*=.338). This indicated that, after accounting for other factors, bullying by itself did not independently affect the likelihood of engaging in sexual intercourse.

Protective factors included sedentary behavior, which reduced odds after adjustment (AOR=0.70, *P*<.001), while parental bonding, attachment, and supervision were significant in unadjusted models but lost significance in adjusted models (*P*>.05). Truancy was a significant factor in both models (AOR=1.57, *P*<.001), highlighting its potential role in outcomes.

## Discussion

The purpose of this study was to investigate the factors that affect adolescent sexual intercourse by addressing socio-demographics, substance use, psychosocial distress, and protective factors. The results of the multivariate analysis showed that while parental bonding, sedentary behavior, parental attachment, and parental supervision demonstrated a protective effect, age, substance use (alcohol and tobacco), alcohol behavior issues, loneliness, anxiety, suicide ideation, suicide attempt, being physically attacked, physical fights, being bullied, and truancy were significantly linked to increased odds of engaging in sexual intercourse.

The findings of our study revealed that adolescents aged 15 or older were significantly more likely to engage in sexual intercourse compared to those aged 14 or younger. According to a study conducted among Ethiopian youth girls, the odds of ESI were 6.81 times higher for those between the ages of 20 and 24 than for those between the ages of 15 and 19.^8^ In Brazil, risky sexual behavior, alcohol consumption, and tobacco use were all more prevalent among older adolescents.^19^

The findings revealed a significant association between substance use and sexual activity. Adolescents who used tobacco, consumed alcohol, and had alcohol-related behavior issues exhibited adjusted odds of engaging in sexual intercourse that were 2.60, 2.09, and 2.08 times higher, respectively, compared to nonusers. In terms of OR, these were 5.70, 3.85, and 4.26 times higher, respectively. According to a study, teens who use drugs or alcohol and have academic difficulties are more likely to engage in risky sexual behaviors.^20^ Based on a study conducted in New Zealand, daily smokers had 1.4 and 3.0 times the likelihood of having an ESI before the age of 16 in comparison to nonsmokers.^21^ A study discovered that substance use behaviors, such as smoking cigarettes, drinking alcohol, and using marijuana, independently increased the risk of engaging in sexual activity.^22^ Based on a study, the odds of substance use were highest among those who reported having sex with partners of both sexes, and the lowest among those who had no sex.^23^ Studies conducted in Taipei, Shanghai, and Hanoi revealed a strong correlation between sexual behavior and risk behaviors, specifically drug use, drinking, and smoking, among 15- to 24-year-olds in both urban and rural areas.^24^ In Brazil, a higher prevalence of risky sexual behavior and unprotected sex was strongly associated with low maternal education and substance use.^25^

The results showed a significant association between sexual activity and psychosocial distress, with adolescents who had anxiety, suicide ideation, and attempted suicide being 1.93 (OR=1.93), 1.74 (OR=1.74), and 1.69 (OR=1.69) times more likely to have sexual intercourse than those who did not have these distress symptoms. A study that examined adolescents aged 12 to 15 found a positive correlation between sexual activity and suicide attempts (pooled OR: 2.12).^26^ Individuals in the United States were 1.15, 1.18, and 1.36 times more likely to experience suicidal ideation, make suicide plans, and attempt suicide, respectively, after their first sexual experience.^27^ In another earlier study, social anxiety was found to raise the risk of unprotected sex among teenagers in Spain.^28^ In Qazvin, Iran, risky behaviors are significantly and favorably predicted by anxiety, depression, having friends who smoke, suicidal thoughts, and strong suicidal ideation in adolescents between the ages of 14 and 19.^29^ Adolescent sexual behaviors in sub-Saharan Africa are associated with alcohol consumption and psychosocial distress, and the risks rise as these factors increase.^30^

The results of this research also showed that adolescents who were bullied had 1.37 (OR=1.37) times higher odds of having sexual intercourse than their counterparts. Globally, teenagers who experienced 20 to 30 days of bullying in the previous month were 2.08, 1.70, and 1.72 times more likely than their peers to report having multiple sexual partners, engaging in sexual activity, and not using a condom, respectively.^31^ A study conducted among Dane County high school students found that risky sexual activity was more common among bullies and bully-victims.^32^ According to a study conducted in Ibadan, Nigeria, 60% of secondary school students said they had engaged in date fighting, and this behavior was significantly associated with sexual risk behaviors like not using a condom, having several sexual partners, and having a history of sexual abuse.^33^ Adolescents who fight physically, motivated by behavioral risks, socioeconomic difficulties, and a lack of parental support, are more likely to engage in risky sexual behaviors.^34^

The findings indicated that adolescents with protective factors such as parental bonding, parental attachment, and parental supervision had 46% (OR=0.54), 30% (OR=0.70), and 36% (OR=0.64) lower odds, respectively, of engaging in sexual intercourse. As reported by a study, parental supervision protected adolescents from risky sexual behaviors and an early sexual debut.^20^ A study conducted in high schools in the United States found that the absence of parental supervision over television programs was associated with a 1.35-fold increased risk of sexual initiation within a year.^35^ In San Francisco, there was a significant decrease in the likelihood of sexual intentions among teenagers between the ages of 14 and 18 who reported having effective parental supervision.^36^ In Kenya, adolescents between the ages of 12 and 19 who communicated with their parents were less likely to transition to their first sexual experience, suggesting that good parent-adolescent communication could act as a buffer against ESI.^37^ The frequency of both protected and unprotected sex among adolescents in Brazil was found to be higher when living with only one or neither parent and when parental supervision was low.^38^ According to an integrative literature review, Asian-American adolescents are more likely to engage in sexually risky behaviors and become pregnant or contract STIs/HIV as a result of family values, parental relationships, acculturation, gender roles, and a lack of sex education.^39^

The findings indicated that adolescents who missed class without permission (truancy) had a 1.57 (AOR=1.57) times higher likelihood of engaging in sexual intercourse compared to their peers who attended class. The GSHS found that adolescents were less likely to participate in risky behaviors and sexual activity if their parents understood their issues, kept monitors on their academic and leisure activities, and respected their privacy.^40^

Adolescent sexual behavior, while a natural aspect of development, can pose serious health and social risks when it occurs without adequate knowledge, emotional maturity, or access to protective resources. Engaging in early or unprotected sexual activity can lead to unintended pregnancies, STIs, and long-term psychosocial challenges. Therefore, schools should introduce age-appropriate, evidence-based sexual education programs that not only provide accurate information but also empower adolescents to make responsible choices. Schools and communities should implement initiatives to reduce adolescent substance abuse by focusing on awareness campaigns, peer mentoring, and providing alternative activities that steer youth away from harmful behaviors. Expanding mental health services in schools, including counseling and support groups, is essential to address emotional distress and reduce the factors contributing to risk-taking behavior. Programs that educate parents about healthy communication, emotional support, and appropriate supervision can foster a positive home environment and reduce adolescent engagement in risky sexual behaviors. Interventions to reduce absenteeism through mentorship, academic support, and attendance incentives can help keep adolescents in structured environments, thus decreasing exposure to risky situations. Promoting physical and recreational activities is another preventive strategy. Encouraging adolescents to participate in sports, arts, and extracurricular activities provides healthy outlets and diverts attention from risky behaviors.

This study on factors associated with sexual behaviors among Uruguayan adolescents presented several significant advantages. It provided a comprehensive analysis by examining a wide range of factors, including socio-demographic characteristics, substance use, psychological distress, and protective factors, offering a holistic understanding of adolescent behavior. The inclusion of both risk and protective factors allowed for a balanced perspective, which could have informed preventive strategies. By focusing on a specific population in Uruguay, the study ensured contextually relevant findings that could be directly applied to local public health initiatives.

The study’s emphasis on substance use and psychological distress highlighted critical areas of intervention and contributed to the identification of vulnerable groups. It also underscored the importance of protective factors such as parental supervision and bonding, offering insights into how family dynamics influenced sexual behavior. The findings helped guide the development of tailored prevention programs targeting high-risk adolescents. The results supported the shaping of policies aimed at promoting safer sexual behaviors and improving overall adolescent health in Uruguay.

The study had several disadvantages. The cross-sectional design limited the ability to establish causal relationships between factors and sexual behavior. The findings might not have been generalizable to adolescents outside of Uruguay due to cultural and contextual differences. Potential confounding variables were not accounted for, which could have influenced the results. The focus on specific protective factors may have excluded other important influences on adolescent sexual behavior. Self-reported data might have introduced biases or inaccuracies, affecting the reliability of the findings. The study also lacked several important parameters that could have shaped the outcomes. School-level effects were not considered, which might have impacted adolescent behaviors due to differing institutional policies and environments. Online relationships were not assessed, even though they likely played a significant role in shaping adolescents’ sexual behavior. The use of social media, which might have shaped attitudes and behaviors, was also not included in the analysis. These missing variables limited the overall depth and comprehensiveness of the study.

## Conclusion

The study highlights several significant factors associated with adolescent sexual behavior. Older adolescents were significantly more likely to engage in sexual activity, indicating age as a key determinant. Substance use, especially tobacco and alcohol, emerged as strong and consistent risk factors. Alcohol-related behavioral issues further increased the likelihood of sexual involvement. Psychological distress, particularly anxiety, also contributed to higher odds of engaging in sexual activity. Involvement in physical fights was associated with increased risk. Truancy stood out as a strong predictor, highlighting the importance of school engagement. While parental bonding, attachment, and supervision initially showed protective effects, they became insignificant after adjustment, suggesting other variables had a stronger influence. Sedentary behavior was found to lower the likelihood of sexual activity. These findings suggested that adolescent sexual behavior was shaped by a combination of behavioral, emotional, and social factors.

## CRediT authorship contribution statement

**Tonmoy Alam Shuvo:** Conceptualization, Formal analysis, Methodology, Supervision, Visualization, Writing – original draft, Writing – review & editing. **Kabir Hossain:** Conceptualization, Investigation, Methodology, Software, Validation, Visualization, Writing – original draft, Writing – review & editing. **Arifur Rahman:** Software, Validation, Writing – original draft, Writing – review & editing.

## Appendix Supplementary materials

Image, application 1

## Footnotes

Supplementary material associated with this article can be found in the online version at doi:10.1016/j.xagr.2025.100538.

## References

[1] Sanci L., Webb M., Hocking J. (2018). Risk-taking behaviour in adolescents. Aust J Gen Pract.

[2] Alamrew Z., Bedimo M., Azage M. (2013).

[3] Watanabe S. (2010). Asymptotic equivalence of Bayes cross validation and widely applicable information criterion in singular learning theory. J Mach Learn Res.

[4] Sexual Risk Behaviors | Reducing Health Risks Among Youth | CDC. Accessed Dec. 24, 2024. https://www.cdc.gov/youth-behavior/risk-behaviors/sexual-risk-behaviors.html

[5] Jing Z., Li J., Wang Y., Zhou C. (2023). Prevalence and trends of sexual behaviors among young adolescents aged 12 years to 15 years in low and middle-income countries: population-based study. JMIR Public Heal Surveill.

[6] Leung J., Lim C., Belete H., Mcclure-Thomas C., Foo S., Chan GCK. (2024). Regional and country prevalence estimates of unsafe sex among adolescents in 68 low-income and middle-income countries. Arch Sex Behav.

[7] Monte L.L., Rufino A.C., Madeiro A. (2024). Prevalence and factors associated with risky sexual behavior among Brazilian school adolescents. Cienc e Saude Coletiva.

[8] Abate B.B., Kassie A.M., Kassaw MW. (2020). Prevalence and determinants of early initiation of sexual intercourse among youth girls in Ethiopia. J Pediatr Nurs.

[9] Lammers C., Ireland M., Resnick M., Blum R. (2000). Influences on adolescents’ decision to postpone onset of sexual intercourse: a survival analysis of virginity among youths aged 13 to 18 years. J Adolesc Heal.

[10] Caminis A., Henrich C., Ruchkin V., Schwab-Stone M., Martin A. (2008). Psychosocial predictors of sexual initiation and high-risk sexual behaviors in early adolescence. Child Adolesc Psychiatry Ment Health.

[11] Turi E., Merga B.T., Fekadu G., Abajobir AA. (2020). Why too soon? Early initiation of sexual intercourse among adolescent females in Ethiopia: evidence from 2016 Ethiopian demographic and health survey. Int J Womens Health.

[12] Efrati Y. (2024). Parental practices as predictors of adolescents’ compulsive sexual behavior: a 6-month prospective study. Eur Child Adolesc Psychiatry.

[13] King R.A., Schwab-Stone M., Flisher A.J. (2001). Psychosocial and risk behavior correlates of youth suicide attempts and suicidal ideation. J Am Acad Child Adolesc Psychiatry.

[14] Benchamas J., Senahad N., Padchasuwan N.H., Laoraksawong P., Phimha S., Banchonhattakit P. (2024). Factors associated with risky sexual behaviors among undergraduate students in Thailand. BMC Public Health.

[15] Reis L.F., Surkan P.J., Atkins K., Garcia-Cerde R., Sanchez ZM. (2023). Risk factors for early sexual intercourse in adolescence: a systematic review of cohort studies. Child Psychiatry Hum Dev.

[16] Pengpid S., Peltzer K., Rodríguez MJ. (2024). Adolescent health-risk behaviours in Uruguay: patterns from national cross-sectional school surveys conducted in 2006, 2012 and 2019. Trop Med Int Heal.

[17] Uruguay—Global School-Based Student Health Survey (2019). https://extranet.who.int/ncdsmicrodata/index.php/catalog/945.

[18] Noncommunicable Disease Surveillance, Monitoring and reporting. Accessed May 28, 2025. https://www.who.int/teams/noncommunicable-diseases/surveillance/systems-tools/global-school-based-student-health-survey

[19] de Moura L.R., Torres L.M., Cadete M.M.M., Cunha C., de F. (2018). Factors associated with health risk behaviors among Brazilian adolescents: an integrative review. Rev da Esc Enferm.

[20] Lohman B.J., Billings A. (2008). Protective and risk factors associated with adolescent boys’ early sexual debut and risky sexual behaviors. J Youth Adolesc.

[21] Paul C., Fitzjohn J., Herbison P., Dickson N. (2000). The determinants of sexual intercourse before age 16. J Adolesc Heal.

[22] Cavazos-Rehg P.A., Krauss M.J., Spitznagel E.L., Schootman M., Cottler L.B., Bierut LJ. (2011). Substance use and the risk for sexual intercourse with and without a history of teenage pregnancy among adolescent females. J Stud Alcohol Drugs.

[23] Brewster K.L., Tillma KH. (2012). Sexual orientation and substance use among adolescents and young adults. Am J Public Health.

[24] Tu X., Lou C., Gao E., Li N., Zabin LS. (2012). The relationship between sexual behavior and nonsexual risk behaviors among unmarried youth in Three Asian Cities. J Adolesc Heal.

[25] Oliveira-Campos M., Nunes M.L., Madeira F. (2014). Comportamento sexual em adolescentes Brasileiros, Pesquisa nacional de Saúde do Escolar (PeNSE 2012). Rev Bras Epidemiol.

[26] Smith L., Jackson S.E., Vancampfort D. (2020). Sexual behavior and suicide attempts among adolescents aged 12–15 years from 38 countries: a global perspective. Psychiatry Res.

[27] Baiden P., Panisch L.S., Kim Y.J., Labrenz C.A., Kim Y., Onyeaka HK. (2021). Association between first sexual intercourse and sexual violence victimization, symptoms of depression, and suicidal behaviors among adolescents in the united states: findings from 2017 and 2019 national youth risk behavior survey. Int J Environ Res Public Health.

[28] Carcedo R.J., Fernández-Rouco N., Fernández-Fuertes A.A., Martínez-álvarez J.L. (2020). Association between sexual satisfaction and depression and anxiety in adolescents and young adults. Int J Environ Res Public Health.

[29] Soleimani M.A., Pahlevan Sharif S., Bahrami N., Yaghoobzadeh A., Allen K.A., Mohammadi S. (2019). The relationship between anxiety, depression and risk behaviors in adolescents. Int J Adolesc Med Health.

[30] Page R.M., Hall CP. (2009). Psychosocial distress and alcohol use as factors in adolescent sexual behavior among sub-Saharan African adolescents. J Sch Health.

[31] Smith L., Grabovac I., Jacob L. (2020). Bullying victimization and sexual behavior among adolescents aged 12–15 years from 53 countries: a global perspective. J Sex Med.

[32] Holt M.K., Matjasko J.L., Espelage D., Reid G., Koenig B. (2013). Sexual risk taking and bullying among adolescents. Pediatrics.

[33] Olley BO. (2015). Date fighting and sexual risk behaviours among adolescents attending public schools in Ibadan, Nigeria. Gend Behav.

[34] Pengpid S., Peltzer K. (2020).

[35] Ashby S.L., Arcari C.M., Edmonson MB. (2006). Television viewing and risk of sexual initiation by young adolescents. Arch Pediatr Adolesc Med.

[36] Sieverding J.A., Adler N., Witt S., Ellen J. (2005). The influence of parental monitoring on adolescent sexual initiation. Arch Pediatr Adolesc Med.

[37] Okigbo C.C., Kabiru C.W., Mumah J.N., Mojola S.A., Beguy D. (2015). Influence of parental factors on adolescents’ transition to first sexual intercourse in Nairobi, Kenya: a longitudinal study. Reprod Health.

[38] Oliveira-Campos M., Giatti L., Malta D., Barreto SM. (2013). Contextual factors associated with sexual behavior among Brazilian adolescents. Ann Epidemiol.

[39] Lee Y.M., Florez E., Tariman J., McCarter S., Riesche L. (2015). Factors related to sexual behaviors and sexual education programs for Asian-American adolescents. Appl Nurs Res.

[40] Shawon M.S.R., Huda N.N., Rouf R.R., Hossain F.B., Al Kibria GM (2024). Associations of parents-adolescent relationship with adolescent sexual risk behaviors: a global analysis based on 156,649 school-going adolescents from 50 countries. Int J Sex Heal.

