# Supplementary material for: Sexual behavior and its influencing factors among Uruguayan adolescents: a cross-sectional study
Source: AJOG Glob Rep. 2025 Jun 19;5(3):100538. doi: 10.1016/j.xagr.2025.100538 (PMC12296454; doi:10.1016/j.xagr.2025.100538)
Supplement: Supplementary file 1 [file mmc1.docx]

**Mixed-effects logistic regression model**

A mixed-effects logistic regression model is used to analyze binary outcomes while accounting for both fixed and random effects. Fixed effects estimate the overall relationship between predictors and the outcome, while random effects account for clustering or group-level variation. This model is especially useful when data are nested, such as individuals within schools or communities. The general equation of the mixed-effect logistic regression model is as follows:

logit(P(Yij​=1)) =β_0_ ​+β_1_​X_1_ij​+β_2_ X_2_ij​+⋯+βpXpij​+U_0j_​

Yij​: Binary outcome variable (0 or 1) for individual i in group/cluster j

P(Yij=1): Probability that the outcome is 1

β_0_​: Fixed intercept (overall average log-odds)

β_1_, β_2_ ,..., βp​: Fixed effect coefficients for predictors X_1_ij, X_2_ij ,..., Xpij

U_0j_​: Random intercept for cluster j, often assumed to be normally distributed.

**Odds Ratio**

Odds ratios were estimated using mixed-effects logistic regression models. Each model included one independent variable at a time, with sexual intercourse as the dependent variable, measured as a binary outcome. A random intercept was included to account for clustering effects across groups. The models were as follows:

**Socio-demographics**

**Gender:**

logit(P(Yij​=1)) =β0​+β1​.Genderij​+U_0j​_

**Age:**

logit(P(Yij​=1)) =β0​+β1​⋅Ageij+U_0j​_

**Hunger:**

logit(P(Yij​=1)) =β0​+β1​.Hungerij​+U_0j​_

**Substance use**

**Tobacco Use:**

logit(P(Yij=1)) =β0+β1⋅Tobaccoij+U_0j_

**Alcohol Use:**

logit(P(Yij=1)) =β0+β1⋅Alcoholij+U_0j_

**Alcohol Behavior Issue:**

logit(P(Yij=1)) =β0+β1⋅AlcoholBehaviorIssueij+U_0j_

**Psychosocial distress**

**Loneliness:**

logit(P(Yij=1)) =β0+β1⋅Lonelinessij+U_0j_

**Anxiety:**

logit(P(Yij=1)) =β0+β1⋅Anxietyij+U_0j_

**Suicide Ideation:**

logit(P(Yij=1)) =β0+β1⋅SuicideIdeationij+U_0j_

**Suicide Attempt:**

logit(P(Yij=1)) =β0+β1⋅SuicideAttemptij+U_0j_

**Physically Attacked:**

logit(P(Yij=1)) =β0+β1⋅PhysicallyAttackedij+U_0j_

**Physical Fight:**

logit(P(Yij=1)) =β0+β1⋅PhysicalFightij+U_0j_

**Bullied:**

logit(P(Yij=1)) =β0+β1⋅Bulliedij+U_0j_

**Protective factors**

**Close Friend:**

logit(P(Yij​=1)) =β0​+β1​.CloseFriendij​+U_0j_

**Peer Support:**

logit(P(Yij​=1)) =β0​+β1​⋅PeerSupportij​+U_0j_

**Parental Bonding:**

logit(P(Yij=1)) =β0+β1⋅ParentalBondingij+U_0j_

**Sedentary Behavior:**

logit(P(Yij=1)) =β0+β1⋅SedentaryBehaviorij+U_0j_

**Parental Attachment:**

logit(P(Yij=1)) =β0+β1⋅ParentalAttachmentij+U_0j_

**Parental Supervision:**

logit(P(Yij=1)) =β0+β1⋅ParentalSupervisionij+U_0j_

**Others**

**Truancy:**

logit(P(Yij=1)) =β0+β1⋅Truancyij+U_0j_

**For all models,**

Yij​: A binary variable indicating whether individual i in group j **has had sexual intercourse.**

Yij =1: Yes (has had sexual intercourse)

Yij ​=0: No (has not had sexual intercourse)

U_0j_: Random intercept (or random effect) for cluster/group j

**Adjusted Odds Ratio**

Adjusted odds ratios (AORs) were estimated using a mixed-effects logistic regression model. All independent variables were included simultaneously to assess their association with sexual intercourse while controlling for other factors. A random intercept was incorporated to account for clustering within groups. The model was as follows:

logit(P(Yij​=1))​=β0​+β1​⋅Ageij​+β2​⋅Genderij​+β3​⋅Hungerij​+β4​⋅Tobaccoij​+β5​⋅Alcoholij​+β6​⋅AlcoholBehaviorIssueij​+β7​⋅Lonelinessij​+β8​⋅Anxietyij​+β9​⋅SuicideIdeationij​+β10​⋅SuicideAttemptij​+β11​⋅PhysicalFightij​+β12​⋅Bulliedij​+β13​⋅PhysicallyAttackedij​+β14​⋅CloseFriendij​+β15​⋅PeerSupportij​+β16​⋅ParentalSupervisionij​+β17​⋅ParentalAttachmentij​+β18​⋅ParentalBondingij​+β19​⋅SedentaryBehaviorij​+β20​⋅Truancyij​+U_0j_

**Where,**

Yij​: A binary variable indicating whether individual i in group j **has had sexual intercourse.**

Yij =1: Yes (has had sexual intercourse)

Yij ​=0: No (has not had sexual intercourse)

U_0j_​: Random intercept (or random effect) for cluster/group j
